# Primary respiratory disease in patients with systemic lupus erythematosus: data from the Spanish rheumatology society lupus registry (RELESSER) cohort

**DOI:** 10.1186/s13075-018-1776-8

**Published:** 2018-12-19

**Authors:** Javier Narváez, Helena Borrell, Fernando Sánchez-Alonso, Iñigo Rúa-Figueroa, Francisco Javier López-Longo, María Galindo-Izquierdo, Jaime Calvo-Alén, Antonio Fernández-Nebro, Alejandro Olivé, José Luis Andreu, Víctor Martínez-Taboada, Joan Miquel Nolla, José María Pego-Reigosa, on behalf of the RELESSER Study Group, Paloma Vela, Elia Vals, Tatiana Cobo-Ibáñez, Gema Bonilla, María Jesús García-Villanueva, Elvira Diez-Álvarez, Mercedes Freire, Marian Gantes, Paloma García de la Peña, Rosario García-Vicuña, José Ángel Hernández-Beiraín, Loreto Horcada, Jesús Ibañez-Ruán, Mónica Ibañez, Carlos Marras, José Luis Marenco, Ivan Castellví, Carlos Montilla-Morales, Mireia Moreno, Ángela Pecondon-Español, Esther Ruiz Lucea, Ana Sánchez-Atrio, Gregorio Santos-Soler, Francisco Toyos, Esther Uriarte Isacelaya, Tomas Vazquez-Rodríguez

**Affiliations:** 1 Department of Rheumatology (Planta 10-2), Servicio de Reumatología, Hospital Universitario de Bellvitge, Feixa Llarga, s/n, Hospitalet de Llobregat, 08907 Barcelona, Spain; 2 Unidad de Investigación, Sociedad Española de Reumatología, Madrid, Spain; 3 Hospital Universitario Doctor Negrín, Las Palmas de, Gran Canaria Spain; 4 Hospital Universitario Gregorio Marañón, Madrid, Spain; 5 Hospital Universitario 12 de Octubre, Madrid, Spain; 6 Hospital Universitario Araba, Vitoria, Spain; 7 Hospital Universitario de Málaga, Málaga, Spain; 8 Hospital Germans Trias i Pujol, Badalona, Barcelona, Spain; 9 Hospital Universitario Puerta de Hierro, Madrid, Spain; 10 Hospital Universitario Marqués de Valdecilla, Santander, Spain; 11 Complexo Hospitalario Universitario de Vigo, Vigo, Spain

**Keywords:** Systemic lupus erythematosus, Pleuropulmonary involvement

## Abstract

**Background:**

The purpose of this study was to assess the prevalence, associated factors, and impact on mortality of primary respiratory disease in a large systemic lupus erythematosus (SLE) retrospective cohort.

**Methods:**

All adult patients in the RELESSER-TRANS (Registry of Systemic Lupus Erythematosus Patients of the Spanish Society of Rheumatology [SER], cross-sectional phase) registry were retrospectively investigated for the presence of primary pleuropulmonary manifestations.

**Results:**

In total 3215 patients were included. At least one pleuropulmonary manifestation was present in 31% of patients. The most common manifestation was pleural disease (21%), followed by lupus pneumonitis (3.6%), pulmonary thromboembolism (2.9%), primary pulmonary hypertension (2.4%), diffuse interstitial lung disease (2%), alveolar hemorrhage (0.8%), and shrinking lung syndrome (0.8%).

In the multivariable analysis, the variables associated with the development of pleuropulmonary manifestation were older age at disease onset (odds ratio (OR) 1.03, 95% confidence interval (CI) 1.02–1.04), higher SLEDAI (Systemic Lupus Erythematosus Disease Activity Index) scores (OR 1.03, 95% CI 1.00–1.07), the presence of Raynaud’s phenomenon (OR 1.41, 95% CI 1.09–1.84), secondary antiphospholipid syndrome (OR 2.20, 95% CI 1.63–2.97), and the previous or concomitant occurrence of severe lupus nephritis, (OR 1.48, 95% CI 1.12–1.95) neuropsychiatric manifestations (OR 1.49, 95% CI 1.11–2.02), non-ischemic cardiac disease (OR 2.91, 95% CI 1.90–4.15), vasculitis (OR 1.81, 95% CI 1.25–2.62), hematological manifestations (OR 1.31, 95% CI 1.00–1.71), and gastrointestinal manifestations, excluding hepatitis (OR 2.05, 95% CI 1.14–3.66). Anti-RNP positivity had a clear tendency to significance (OR 1.32, 95% CI 1.00–1.75; *P* = 0.054).

The development of pleuropulmonary manifestations independently contributes to a diminished survival (hazard ratio of 3.13). However, not all complications will influence the prognosis in the same way. Whereas the occurrence of pleural disease or pulmonary thromboembolism has a minimal impact on the survival of these patients, the remaining manifestations have a major impact on mortality.

**Conclusion:**

Except for pleural disease, the remaining respiratory manifestations are very uncommon in SLE (<4%). Pleuropulmonary manifestations independently contributed to a decreased survival in these patients.

**Electronic supplementary material:**

The online version of this article (10.1186/s13075-018-1776-8) contains supplementary material, which is available to authorized users.

## Background

Primary respiratory disease in systemic lupus erythematosus (SLE) seems not uncommon, although its exact prevalence is still unknown, since it has been reported to occur in 5% to 90% of the patients [1–6]. This wide range in prevalence may be explained by the characteristics of the patients studied (early or established disease), whether only symptomatic patients were included or systematic screening was done, the methodology used for diagnosis (clinical manifestations and imaging or autopsy findings), and the different study designs.

All components of the respiratory system may be affected during the course of disease. The spectrum of pulmonary manifestations caused by SLE includes pleural disease, upper and lower airway dysfunction, primary pulmonary hypertension, pulmonary thromboembolism, acute reversible hypoxemia, diffuse interstitial lung disease, acute lupus pneumonitis, diffuse alveolar hemorrhage, and shrinking lung syndrome [1–16]. Some patients may have more than one form of pleuropulmonary involvement during the course of their disease. The severity of these respiratory complications is highly variable and ranges from subclinical to potentially life-threatening conditions.

In general terms, primary respiratory involvement in SLE is not as well known as other major organ involvement. As in many other aspects of SLE, there is still no clear explanation for the appearance of such different respiratory manifestations in patients with an identical underlying disease. Although some associations have been described [1–6], the risk factors related to the occurrence of these complications are still unknown. It is likely that there are risk factors associated with the development of these complications, some of which may be modifiable.

The appearance of respiratory complications influences the prognosis (functional and vital) and the accumulated damage of the disease, although the extent and grade are still unknown. As the spectrum of pulmonary manifestations caused by SLE itself is very broad, not all complications will influence the prognosis in the same way.

The aim of the present study was to investigate the prevalence, risk factors, and impact on mortality of primary respiratory disease in patients from the multicenter Spanish SLE cohort RELESSER-TRANS (Registry of Systemic Lupus Erythematosus Patients of the Spanish Society of Rheumatology [SER], cross-sectional phase).

## Methods

### Patient selection

This is a historical study in which all adult patients in the RELESSER-TRANS registry were retrospectively investigated for the presence of primary pleuropulmonary manifestations.

The RELESSER-TRANS is a hospital-based registry involving a cross-sectional stage, designed to obtain a better understanding of SLE in clinical settings. It includes data from 3679 SLE patients (American College of Rheumatology [ACR]-1997 criteria) [17] from 45 Spanish hospitals. The methodologic and general characteristics of the RELESSER registry were published previously [18] (Additional file 1).

Pleuropulmonary manifestations analyzed in this study included (1) pleural disease, including patients with episodes of pleurisy according with the SELENA-SLEDAI (Systemic Lupus Erythematosus Disease Activity Index) definition [19], or pleural fibrosis according with the Systemic Lupus International Collaborating Clinics (SLICC)/ACR-damage index (SDI) [20] or both; (2) acute lupus pneumonitis or interstitial alveolitis/pneumonitis (in accordance with the British Isles Lupus Assessment group [BILAG] 2004 definition) [21, 22]; (3) diffuse interstitial lung disease or pulmonary fibrosis (SDI definition); (4) pulmonary hemorrhage (BILAG 2004 definition); (5) shrinking lung syndrome (BILAG 2004 definition); (6) primary pulmonary hypertension (SDI definition); and (7) pulmonary thromboembolism, including the cases registered with pulmonary infarction in accordance with the SDI definition. Upper and lower airway dysfunction and acute reversible hypoxemia were not included in the study as these two manifestations were not specifically recorded in the RELESSER database. Respiratory involvement was considered primary when it was directly related to SLE activity, and infections, drug toxicity, chronic obstructive pulmonary disease, occupational exposure, and neoplasia had been excluded.

The study protocol was approved by the institutional ethics committee of the Hospital Universitario Doctor Negrín and subsequently by the local ethics committee of all participating centers. Informed consent was obtained from the patients, and their clinical records and information were anonymized prior to analysis. Confidential information of the patients was protected in accordance with national norms. This study was conducted in accordance with the principles of the Declaration of Helsinki and the International Conference for Harmonization.

### Statistical analysis

Numerical variables were expressed as mean or median and standard deviation. The categorical variables were described by absolute frequency and percentage.

Comparisons of numerical variables were obtained by using a Student *t* test or a Mann–Whitney *U* test, according to normality adjustments, and of categorical variables by using a chi-squared or Fisher’s exact test as necessary.

Variables reaching statistical significance in the unadjusted analysis were entered in a multivariate model (Cox proportional hazards regression) to identify risk factors for the development of pleuropulmonary complications. The selection of variables in the model was made while taking into account its individual association, the multicollinearity between different variables, and its importance as a confounding factor that justified its inclusion as an adjustment variable.

Survival analysis (Kaplan–Meier) was conducted to assess whether the development of respiratory disease was associated with lower survival in patients with lupus and to explore the influence on the prognosis of each analyzed respiratory manifestation. Subsequent comparisons between survival curves were made by using a log-rank test.

To determine the independent contribution of the presence of pleuropulmonary manifestations to mortality, a multivariable Cox regression analysis was carried out. The following variables were considered candidates for inclusion: age, gender, infections, nephritis, cardiac involvement, cardiovascular and cerebrovascular events, and SLEDAI and SLICC scores. Pleuropulmonary manifestations were considered a time-dependent variable. Statistical significance was assumed as a *P* value of less than 0.05.

## Results

### Demographic data

Of the 3679 patients included in the RELESSER-TRANS, 3215 patients were finally included in this study (after excluding those in whom respiratory manifestations had not been collected and cases in which there were doubts as to whether the pulmonary condition could be directly related to SLE activity). Of these, 91% were female and 9% were male; mean age at SLE diagnosis (± standard deviation) was 37 ± 13 years (range 19–86), and median disease duration was 118 ± 98 months (range 61–196). The main baseline demographic and clinical characteristics of the SLE study cohort are shown in Table 1.

**Table 1:** Baseline demographic and clinical characteristics of the systemic lupus erythematosus study cohort

Demographic characteristics	
Number of patients	3215
Women/Men	2925 (91%)/290 (9%)
Age at SLE diagnosis, mean ± SD	37 ± 13 (range, 19–86 years)
Age at the time of RELESSER-TRANS inclusion, mean ± SD	48 ± 14 (range, 19–94 years)
Ethnic groups	Caucasians: 93%/Hispanics: 5.4%/African-Americans: 0.2%/Asians: 0.6%/Others: 0.8%
Tobacco	Never: 58%/Before: 25%/At the time of RELESSER-TRANS inclusion: 17%
Clinical manifestations	
Constitutional symptoms	Fever 3.6%/Weight loss 9.5%
Cutaneous	64%
Arthritis	78%
Raynaud’s phenomenon	35%
Vasculitis	10%
Nephritis	31%
Gastrointestinal involvement	3%
Hepatitis	2.8%
Pericarditis	15%
Non-ischemic cardiac disease	4.5%
Neuropsychiatric manifestations	6.5%
Hematologic	79%
Lymphadenopathy/Splenomegaly	9.7%/2.9%
Secondary antiphospholipid syndrome	17%
SLEDAI score, mean ± SD	2.62 ± 3.69
SDI score, mean ± SD	1.15 ± 1.68
Immunological laboratory^*^	
Anti-dsDNA antibody positivity (available data in all patients)	72%
Hypocomplementemia (available data in all patients)	76.5%
Anti-Sm antibody positivity (available in 3048 patients)	21%
Anti-Ro antibody positivity (available in 3109 patients)	40%
Anti-La antibody positivity (available in 3108 patients)	19%
Anti-RNP antibody positivity (available in 3095 patients)	25%
Anticardiolipin antibody positivity [IgM or IgG] (available in 2952 patients)	20%
Anti-beta 2 glycoprotein 1 [IgM or IgG] (available in 1918 patients)	13.5%
Lupus anticoagulant (available in 2312 patients)	23%
Treatments^**^	
Corticosteroids	88%
<10 mg/daily of prednisone or equivalent	46.5%
10–30 mg/daily	32%
>30–60 mg/daily	21.5%
Antimalarials	83.5%
Methotrexate	17%
Azathioprine	31%
Cyclophosphamide	20%
Mycophenolate	16%
Intravenous immunoglobulins	4%
Plasmapheresis	1.5%
Rituximab	6%
Antiplatelet therapy	36%
Anticoagulants	14%

Abbreviations: *RELESSER-TRANS* Registry of Systemic Lupus Erythematosus Patients of the Spanish Society of Rheumatology [SER], cross-sectional phase, *SD* standard deviation, *SDI* Systemic Lupus International Collaborating Clinics/American College of Rheumatology Damage Index, *SLE* systemic lupus erythematosus, *SLEDAI* Systemic Lupus Erythematosus Disease Activity Index

^*^Because this is a retrospective (historical) study, not all analyzed variables were recorded in all included cases. Thus, the percentage for each variable was calculated for only those patients in which the data were documented

^**^ Treatments recorded in the RELESSER-TRANS registry as “any use” or “use at last visit” (corresponding to the last visit before enrollment) or both

### Pleuropulmonary manifestations

At least one pleuropulmonary manifestation was present in 996 patients (31%): 920 patients developed one manifestation, 56 two, 19 three, and 1 four. The most common manifestation was pleural disease, occurring in 680 (21%) of patients, followed by lupus pneumonitis in 118 (3.6%), pulmonary thromboembolism in 95 (2.9%), primary pulmonary hypertension in 79 (2.4%), diffuse interstitial lung disease in 65 (2%), alveolar hemorrhage in 28 (0.8%), and shrinking lung syndrome in 28 (0.8%).

Respiratory manifestations may complicate SLE at any time during its course, although they usually occur later in the course of the disease. The mean time from diagnosis of SLE to the first appearance of respiratory manifestations was 5.8 (standard deviation 8.3) years; the mean disease duration for each manifestation was pleurisy 7.6 years (8.6), acute lupus pneumonitis 5.4 years (7.6), diffuse interstitial lung disease/pulmonary fibrosis 7.7 years (10.5), alveolar hemorrhage 5.2 years (7.5), shrinking lung syndrome 6 years (6.8), primary pulmonary hypertension 9.2 years (9.2), and pulmonary thromboembolism 3.9 years (7.9). In 4% of the cases, respiratory manifestations were the presenting symptoms of SLE (including cases of pleurisy, shrinking lung syndrome, and diffuse alveolar hemorrhage). Twenty-two percent of patients with acute lupus pneumonitis (26 out of 118) and 21% of patients with alveolar hemorrhage (6 out of 28) who survived finally developed pulmonary fibrosis.

### Other previous and concomitant clinical manifestations of SLE

As shown in Table 1, the majority of patients who developed pleuropulmonary manifestations also had previous or concomitant involvement of other major organs. The most common clinical features of lupus were arthritis and dermatologic involvement and, less commonly, pericarditis. Nephritis was documented in 39% of the patients, non-ischemic cardiac disease in 16%, neuropsychiatric involvement in 11%, vasculitis in 8%, and gastrointestinal involvement in 5%.

In regard to their autoantibody profiles, 72% of patients tested positive for anti-double stranded DNA antibodies and 76.5% had hypocomplementemia at some time during the course of the disease. Anti-Sm antibodies were positive in 21%, anti-RNP in 25%, and anti-Ro in 40% of the patients in whom this information was available. The frequency of antiphospholipid antibody (aPL) positivity was also remarkable: anticardiolipin antibodies (IgM or IgG) 20%, anti-beta-2 glycoprotein 1 (IgM or IgG) 13.5%, and lupus anticoagulant 23%.

Patients with respiratory complications had higher S-SLEDAI scores (3.65 ± 2.25 versus 2.72 ± 2.43; *P* <0.001) at the last visit (when enrollment in the registry occurred). As expected, the mean SDI scores were also significantly higher in this group than in patients with no pleuropulmonary involvement (2.39 ± 3.34 versus 0.90 ± 1.40; *P* <0.001). Of interest, when lung manifestations were excluded from the SDI, the differences remained (1.57 ± 2.13 versus 0.62 ± 1.22; *P* <0.01). From these data it can be inferred that the development of respiratory complications has an important weighting in the accumulated damage of the disease.

### Factors associated with the development of pleuropulmonary complications

The results of the univariate and multivariate analysis are shown in Table 2. In the multivariate analysis, the variables independently associated with the development of pleuropulmonary manifestation were older age at disease onset (odds ratio (OR) 1.03, 95% confidence interval (CI) 1.02–1.04), higher SLEDAI scores (OR 1.03, 95% CI 1.00–1.07), the presence of Raynaud’s phenomenon (OR 1.41, 95% CI 1.09–1.84), secondary antiphospholipid syndrome (OR 2.20, 95% CI 1.63–2.97), and the previous or concomitant occurrence of severe lupus nephritis (including classes III, IV, V, and mixed III/IV + V) (OR 1.48, 95% CI 1.12–1.95), neuropsychiatric manifestations (OR 1.49, 95% CI 1.11–2.02), non-ischemic cardiac disease (OR 2.91, 95% CI 1.90–4.15), vasculitis (OR 1.81, 95% CI 1.25–2.62), hematological manifestations (OR 1.31, 95% CI 1.00–1.71), and gastrointestinal manifestations, excluding hepatitis (OR 2.05, 95% CI 1.14–3.66). Anti-RNP positivity had a clear tendency to significance (OR 1.32, 95% CI 1.00–1.75; *P* = 0.054).

**Table 2:** Variables associated with the development of pleuropulmonary manifestations

	Without pleuropulmonary involvement (*N* = 2219)	With pleuropulmonary involvement (*N* = 996)	Univariate analyses	Multivariate analyses	
			*P* value	Odds ratio (95% CI)	*P* value
Gender, female (%)	91.5% (2030)	88.3% (880)	0.053	0.86 (0.57–1.29)	0.450
Age at SLE onset, mean ± SD	37 ± 11.6	39.1 ± 13	**<0.001**	1.03 (1.02–1.04)	**0.001**
Tobacco (before or at the time of RELESSER-TRANS inclusion)	41.4% (919)	45.2% (450)	**0.015**	1.13 (0.88–1.45)	0.350
Clinical manifestations					
Arthritis	77.3% (1717)	80% (797)	0.257	1.35 (0.99–1.85)	0.069
Cutaneous	64.9% (1440)	62.6% (623)	0.568		
Raynaud’s phenomenon	32.6% (723)	41.9% (417)	**<0.001**	1.41 (1.09–1.84)	**0.015**
Vasculitis	7.2% (160)	17.6% (175)	**<0.001**	1.81 (1.25–2.62)	**0.002**
Nephritis	26.8% (595)	39% (389)	**0.043**	1.30 (0.98–1.73)	0.068
Severe lupus nephritis (only including classes III, IV, V, and mixed III/IV + V)	20.2% (450)	26.7% (266)	**<0.001**	1.48 (1.12–1.95)	**0.002**
Non-ischemic cardiac disease, excluding pericarditis	2.3% (52)	9.3% (93)	**<0.001**	2.91 (1.90–4.15)	**0.001**
Gastrointestinal involvement, except hepatitis	2.2% (49)	5.3% (53)	**0.001**	2.05 (1.14–3.66)	**0.016**
Hepatitis	2.3% (51)	3.9% (39)	0.078	0.75 (0.35–1.60)	0.453
Hematologic abnormalities	76.7% (1701)	84.3% (840)	**0.001**	1.31 (1.00–1.71)	**0.048**
Neuropsychiatric manifestations	4.4% (98)	(112)	**<0.001**	1.49 (1.11–2.02)	0.009
Secondary antiphospholipid syndrome	12% (266)	27.9% (278)	**<0.001**	2.20 (1.63–2.97)	**0.000**
SLEDAI score, mean ± SD	2.72 ± 2.43	3.65 ± 2.25	**<0.001**	1.03 (1.00–1.07)	**0.021**
SDI score, mean ± SD	0.90 ± 1.40	2.39 ± 3.34	**<0.001**		
Immunological laboratory*					
Anti-dsDNA antibody positivity	71.3% (1978/2772)	76.7% (277/361)	0.072		
Hypocomplementemia	76.2% (2123/2785)	77.9% (278/357)	0.085		
Anti-Sm antibody positivity	20.2% (545/2701)	23.6% (82/347)	0.134		
Anti-Ro antibody positivity	40.8% (1126/2760)	36.1% (126/349)	0.092		
Anti-La antibody positivity	19.7% (543/2756)	16.5% (58/352)	0.149		
Anti-RNP antibody positivity	23.9% (657/2744)	30.5% (107/351)	**0.007**	1.32 (1.00–1.75)	0.054
Anticardiolipin antibody positivity (IgM or IgG)	19.9% (520/2616)	21.7% (73/336)	0.426		
Anti-beta 2 glycoprotein 1 (IgM or IgG)	13.3% (226/1697)	14.9% (33/221)	0.509		
Lupus anticoagulant	22.2% (455/2049)	30.1% (82/272)	**0.004**		

Results are presented as percentage (and number of cases) or mean ± standard deviation

*Abbreviations*: *CI* confidence interval, *RELESSER-TRANS* Registry of Systemic Lupus Erythematosus Patients of the Spanish Society of Rheumatology [SER], cross-sectional phase, *SD* standard deviation, *SDI* Systemic Lupus International Collaborating Clinics/American College of Rheumatology Damage Index, *SLE* systemic lupus erythematosus, *SLEDAI* Systemic Lupus Erythematosus Disease Activity Index

^*^ Because this is a retrospective (historical) study, not all analyzed variables were available in all included cases. Thus, the percentage for each variable was calculated for only those patients in which the data were documented

Statistical significance was assumed as a *p* value of less than 0.05 (bold data)

### Mortality

**Fig. 1:**
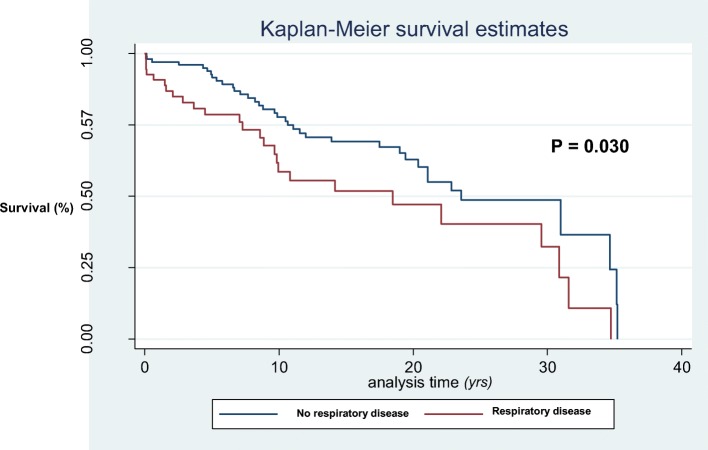
Survival analysis (Kaplan–Meier) comparing patients with and without primary respiratory disease

Sixty-one patients with pleuropulmonary manifestations (1.89%) died over the follow-up period. SLE was the commonest cause of death, occurring in 0.9% of patients (28 out of 3215), followed by infection in 0.5% (17 out of 3215), cardiovascular and cerebrovascular disease in 0.3% (11 out of 3215), and cancer in 0.1% (5 out of 3215). Although the mortality rate was low, the development of respiratory disease was associated with lower survival (survival rates 95.6% versus 82.2%, *P* = 0.030; Fig. 1). After adjustment for known confounders in the multivariable Cox regression model (Table 3), pleuropulmonary manifestations remained a risk factor for diminished survival (hazard ratio 3.13, 95% CI 1.56–6.28, *P* = 0.001).

**Table 3:** Cox regression of predictors of mortality in lupus patients from the RELESSER-TRANS cohort

	Hazard ratio	95% confidence interval	*P* value
Pleuropulmonary manifestations	3.13	1.56–6.28	0.001
Age	0.99	0.96–1.01	0.293
Gender	0.89	0.31–2.52	0.825
Infections	0.79	0.39–1.59	0.506
Nephritis	0.74	0.23–2.39	0.612
Cardiac disease	1.01	0.42–2.42	0.978
Cardiovascular events	1.57	0.32–7.66	0.577
SLEDAI score	1.08	1.00–1.16	0.052
SLICC score	1.79	0.69–0.92	0.002

*Abbreviations*: *RELESSER-TRANS* Registry of Systemic Lupus Erythematosus Patients of the Spanish Society of Rheumatology [SER], cross-sectional phase, *SLEDAI* Systemic Lupus Erythematosus Disease Activity Index, *SLICC* Systemic Lupus International Collaborating Clinics

Since not all complications influenced the prognosis in the same way, survival analysis analyzing the presence of each individual respiratory manifestation was conducted: (1) considering only the presence of pleural disease: survival rates of 82.9% versus 95.6% (no pleuritis) (*P* = 0.101); (2) considering only the presence of pulmonary thromboembolism: survival rates of 88.7% versus 95.6% (*P* = 0.253); (3) considering only the presence of shrinking lung syndrome: survival rates of 80.9% versus 95.6% (*P* = 0.567); (4) considering only the presence of diffuse interstitial lung disease: survival rates of 79.7% versus 95.6% (*P* <0.01); (5) considering only the presence of pneumonitis: survival rates of 78.6% versus 95.6% (*P* <0.01); (6) considering only the presence of primary pulmonary hypertension: survival rates of 76.1% versus 95.6% (*P* <0.01); and (7) considering only the presence of alveolar hemorrhage: survival rates of 55.6% versus 95.6% (*P* <0.001).

## Discussion

The heterogeneity of disease course and outcome in SLE, coupled with its low prevalence, make it difficult for physicians to acquire sufficient clinical experience in the absence of standardization and collaborative efforts. Therefore, much of the clinical research on SLE has been based primarily on registries and on their derived cohorts, which have been an important source of new knowledge about the disease. Studies derived from registries usually have a large number of patients from non-experimental clinical settings and allow a more extensive follow-up than can be accomplished in clinical trials, providing more reliable answers to specific questions. In this case, this applied to primary respiratory involvement in SLE, which is not as well known as other major organ involvement.

According to the data from our registry, the overall prevalence of symptomatic pleuropulmonary manifestations in this SLE population was 31%, and pleural involvement was the most frequent complication. This prevalence is higher than that reported in the Eurolupus project (7%) [23] and is similar to that reported in the Latin American SLE cohort (Grupo Latinoamericano de Estudio del Lupus, or GLADEL), which recently examined this specific clinical aspect (28%) [2]. Prevalence rates from these three multicenter registries demonstrate that primary respiratory disease occurs in about 25–30% of patients with lupus during the course of the disease.

Apart from pleural disease that occurs in 21% of patients, the other respiratory manifestations are uncommon, occurring in less than 4% of cases. Of the lung parenchymal manifestations, the most commonly observed was pneumonitis (3.6%). This is consistent with observations from other registries in which it was also the most frequent parenchymal complication; the reported prevalence was between 2.4% and 4% [2, 24].

Pleuropulmonary involvement may complicate SLE at any time during its course, although it is more frequent later in the course of the disease (mean disease duration of about 6 years). Data from the LUMINA (Lupus in minorities: nature versus nurture) multiethnic cohort also documented the development of pulmonary damage (as defined by SDI) after a mean disease duration of 5.3 years [13]. More rarely (4% of cases in our study), respiratory complications may be the presenting manifestation of SLE. This has been described with pleurisy, shrinking lung syndrome, diffuse alveolar hemorrhage, acute lupus pneumonitis, and pulmonary thromboembolism [14, 16, 24, 25].

In most patients, pleuropulmonary involvement occurs in the setting of previous or concomitant involvement of other major organs. Apart from the frequent presence of systemic, cutaneous, and musculoskeletal symptoms, a considerable proportion of these patients have or have had nephritis (39% in our series and 40% of cases in the GLADEL registry), neuropsychiatric involvement (11% and 14%, respectively), and non-ischemic heart disease (16% and 14%, respectively) [2]. According to our study, the history of pericarditis, vasculitis, and gastrointestinal involvement is also frequent in this group of patients. From these data it can be inferred that the development of respiratory complications occurs in patients with a severe disease, with higher mean SDI scores even when lung manifestations are excluded.

In fact, patients with active disease (defined by higher SLEDAI scores) and the previous or concomitant presence of severe lupus nephritis, neuropsychiatric manifestations, non-ischemic cardiac disease, hematological manifestations, and gastrointestinal involvement, those with Raynaud’s phenomenon, and those who had secondary antiphospholipid syndrome are predisposed to the occurrence of pleuropulmonary manifestations. Another predictive factor is older age. It cannot be ruled out that, in addition to antiphospholipid antibodies, anti-RNP antibodies could be related to the development of pleuropulmonary manifestations; in our study, anti-RNP positivity had a clear tendency to significance (OR 1.32, 95% CI 1.00–1.75; *P* = 0.054) and previous investigations support this relationship.

Some of these associated factors are coincident with those reported in other studies. In the LUMINA registry, older age and the presence of anti-RNP antibodies were associated with a shorter time to the development of permanent lung disease [13]. In the GLADEL registry, older age and the presence of systemic symptoms, non-ischemic heart disease, and nephritis were also associated with the occurrence of pleuropulmonary manifestations [2].

A strong association between aPL and pulmonary thromboembolism has been well documented in SLE [26]. In addition, new evidence suggests that aPL in patients with SLE increases the risk of primary pulmonary hypertension [27]. Hypotheses regarding the impact of aPL on pulmonary hypertension include large vessel and microvascular thrombosis and endothelial remodeling [27]. Diffuse alveolar hemorrhage may rarely occur in antiphospholipid syndrome and may be the initial manifestation [28]. The proposed mechanism in SLE is a necrotizing microangiitis related to immune complex deposition and induction of apoptosis, involving alveolar capillaries [29]. In antiphospholipid syndrome, histopathological studies show alveolar hemorrhage and microvascular thrombosis with or without pulmonary capillaritis [30]. The fact that SLE and antiphospholipid syndrome have a wide spectrum of pulmonary manifestations, some of which may be very similar, has raised the question of common pathological processes [31]. Finally, a greater frequency of Raynaud’s phenomenon in patients with SLE and primary pulmonary hypertension has been documented (60% versus 20%–30% in those without this complication) [32]. Shen et al. have shown that patients with pulmonary hypertensive SLE had higher serum endothelin levels, their lupus was more active, and they presented with Raynaud’s phenomenon, suggesting that pulmonary arterial vasospasm could play an important role in the pathogenesis of this complication [33]. Finally, Wang et al., in a recently published meta-analysis, identified the anti-RNP antibody and anti-Sm antibody as risk factors for SLE-associated pulmonary arterial hypertension with pooled ORs of 3.68 (95% CI 2.04–6.63, *P* <0.0001) and 1.71 (95% CI 1.06–2.76, *P* = 0.03), respectively [34]. Seropositivity for anti-RNP has also been described as a risk factor for the development of shrinking lung syndrome but this association has not been confirmed in all of the studies [16, 35, 36]. Previous investigations have documented a relationship between anti-Ro, anti-La, and anti-Sm antibodies with some pulmonary manifestations, but we could not corroborate these associations [2, 37–39].

Primary respiratory disease is an important cause of morbidity and mortality in SLE. In a previous study from our group, pleuropulmonary manifestations represented 3.7% of the total accumulated damage of the disease in the patients included in the RELESSER-TRANS as defined by the SDI [40]. As stated in the LUMINA cohort, cumulative rates of pulmonary damage at 5 and 10 years are 7.6% and 11.6%, respectively [13]. Another important finding is that 21%–22% of our patients with acute lupus pneumonitis or alveolar hemorrhage who survived finally developed pulmonary fibrosis.

Consistent with previous reports, our study corroborates the fact that pleuropulmonary manifestations independently contribute to a diminished survival [2, 41, 42]. However, not all complications will influence the prognosis in the same way. Whereas the occurrence of pleural disease or pulmonary thromboembolism has a minimal impact on the survival of these patients, the remaining manifestations have a major impact on mortality (in order of least to most important: shrinking lung syndrome, diffuse interstitial lung disease, pneumonitis, primary pulmonary hypertension, and alveolar hemorrhage).

This is a retrospective (historical) study carved into an existing cohort and has several limitations. First, the main limitation is that the baseline variables were collected many years into the course of the disease rather than at onset. Second, owing to the multipurpose nature of the RELESSER-TRANS registry, which was never specifically designed to study primary respiratory disease in SLE, pleuropulmonary manifestations were not systematically evaluated, and only the symptomatic cases were collected. This could lead to an underestimation of the frequency of some of these manifestations. Third, not all autoantibodies were available in all patients, and had not been obtained at a central laboratory or at the time pleuropulmonary manifestations occurred. Fourth, we could not exclude random associations due to the large number of variables analyzed. However, we have tried to minimize this risk by using multivariate analysis. Fifth, the main objectives of this registry did not include analysis of the specific effects of therapies on individual manifestations. For this reason, in the multivariable Cox regression model of mortality, we have not included medications known to be either protective (antimalarials) or deleterious (steroids).

## Conclusions

Except for pleural disease, the remaining respiratory manifestations are very uncommon in patients with SLE, occurring in less than 4% of cases. Of the lung parenchymal manifestations, the most commonly observed was pneumonitis (3.6%).

They occur later in the course of the disease, mainly in patients with active and severe lupus (with previous or concomitant major organ involvement other than lung), and seem to be associated with older age, the presence of Raynaud’s phenomenon, and the positivity of antiphospholipid antibodies. Pleuropulmonary manifestations contribute independently to a significantly decreased survival. However, not all complications will influence the prognosis in the same way. Whereas the occurrence of pleural disease or pulmonary thromboembolism has a minimal impact on the survival of these patients, the remaining manifestations have a major impact on mortality.

## Additional file

Additional file 1: Information about the RELESSER-TRANS registry. (DOCX 15 kb)

## Abbreviations

ACR: American College of Rheumatology
aPL: Antiphospholipid antibody
BILAG: British Isles Lupus Assessment group
CI: Confidence interval
GLADEL: Grupo Latinoamericano de Estudio del Lupus
LUMINA: Lupus in minorities: nature versus nurture
OR: Odds ratio
RELESSER: Registry of Systemic Lupus Erythematosus Patients of the Spanish Society of Rheumatology
RELESSER-TRANS: Registry of Systemic Lupus Erythematosus Patients of the Spanish Society of Rheumatology [SER], cross-sectional phase
SDI: Systemic Lupus International Collaborating Clinics (SLICC)-Damage Index
SELENA-SLEDAI: Safety of Estrogens in Lupus Erythematosus National Assessment-Systemic Lupus Erythematosus Disease Activity Index
SLE: Systemic lupus erythematosus
SLEDAI: Systemic Lupus Erythematosus Disease Activity Index
SLICC: Systemic Lupus International Collaborating Clinics
