# Supplementary material for: Primary respiratory disease in patients with systemic lupus erythematosus: data from the Spanish rheumatology society lupus registry (RELESSER) cohort
Source: Arthritis Res Ther. 2018 Dec 19;20:280. doi: 10.1186/s13075-018-1776-8 (PMC6299951; doi:10.1186/s13075-018-1776-8)
Supplement: Supplementary file 1 — Information about the RELESSER-TRANS registry. (DOCX 15 kb) [file 13075_2018_1776_MOESM1_ESM.docx]

**Information about RELESSER-TRANS registry.**

Members of the Systemic Autoimmune Diseases Study Group of the Spanish Society of Rheumatology (SER), with the methodological support and supervision of the Research Unit of the SER, established the registry of systemic lupus erythematosus of the SER (RELESSER).

RELESSER is a multicenter registry of patients with systemic lupus erythematosus (SLE) which consists of 2 phases, a transversal one (RELESSER-T), for which the inclusion of patients has been already completed, and a longitudinal prospective study conducted on a selected sample of patients included in an initial cohort (RELESSER-PROS).

The first stage, or RELESSER-TRANS (T), is a multicenter, hospital-based registry, with retrospective cross-sectional collection of data from a large representative sample of non-selected adult patients with SLE attending Spanish rheumatology services within the public national health system. Forty-five centers were involved, and all participating researchers received specific training on the study procedures and the use of SLE assessment tools (i.e., activity, severity, and damage indexes). A total of 359 variables per patient were collected, with highly standardized definitions encompassing sociodemographic data, cumulative clinical and laboratory characteristics, as well as comorbidities and Charlson index.

These variables were retrospectively recorded until the last available evaluation by the rheumatologist, or until the patient died. Systemic lupus international collaborating clinics (SLICC)/American College of Rheumatology damage index (SDI), Severity Katz index (SKI)^(1)^, and the SELENA-SLEDAI [S-SLEDAI] activity index at the last visit (when enrollment occurred) were calculated. In order to be included in RELESSER, all patients were required to have recorded at least 50% of those variables. Different procedures were followed to minimize missing data and to ensure data quality, management, and security. The percentage of missing data was < 5% in 92% of the variables collected.

The patients were consecutively included until the end date of the study. The first patient was included on 27-10-2011 and the last on 13- 08-2012, with effective inclusion having a duration of 10 months.

The research unit of the SER managed all data and data processing. This unit was the coordinating center, providing expert methodological support at all stages of the project, carrying out study monitoring and identifying potential inconsistencies and solutions. The research unit of the SER has given expert methodological support to recognized registries of patients with different rheumatic diseases^(2-5)^.

Treatment with glucocorticoids (GC), immunosuppressants (IS), or other therapies was recorded as follows: *“never”, “any use,*” and *use at last visit”.* In the case of antimalarials, time of exposure (months) was also recorded. The data collected in medical records did not specify whether the GC and/or IS were specifically indicated for treatment of the respiratory complication.

Ethnicity was classified as: (1) Caucasians (patients with white European ancestral origins); (2) Hispanics (patients treated in hospitals included in RELESSER and residents in Spain when the study was done, but who were originally from Spanish-speaking countries of Central and South America); (3) Afro-Americans; (4) Asians; and (5) others.

Only severe infections (those requiring hospitalization or cause of death), were collected.

The study protocol has been approved by the institutional ethics committee of the Hospital Universitario Doctor Negrín (Las Palmas de Gran Canaria) and subsequently by the local ethics committee of all participating centers. Informed consent was not obtained from the patients, but their clinical records and information were anonymized prior to analysis. This study was conducted in accordance with the principles of the Declaration of Helsinki and the International Conference for Harmonization.

**References.**

1. Katz JD, Senecal JL, Rivest C, Goulet JR, Rothfield N. A simple severity of disease index for systemic lupus erythematosus. Lupus1993;2:119-23.

2. Carmona L, Ballina J, Gabriel R, Laffon A;  EPISER Study Group. The burden of musculoskeletal diseases in the general population of Spain: results from a national survey. Ann Rheum Dis 2001;60:1040-5.

3. Gonzalez-Alvaro I, Carmona L, Balsa A, Sanmarti R, Belmonte MA, Tena X; EMECAR Study Group. Patterns of disease modifying antirheumatic drug use in a Spanish cohort of patients with rheumatoid arthritis.  J Rheumatol 2003; 30:697–704.

4. Sanchez-Piedra C, Hernández Miguel MV, Manero J, Roselló R, Sánchez-Costa JT, Rodríguez-Lozano C et al; Grupo de trabajo BIOBADASER Fase III. Objectives and methodology of BIOBADASER phase III. Reumatol Clin. 2017 Sep 18. pii: S1699-258X(17)30225-5.

5. Gómez-Reino JJ, Rodríguez-Lozano C, Campos-Fernández C, Montoro M, Descalzo MÁ, Carmona L; BIOBADASER 2.0 Study Group. Change in the discontinuation pattern of tumour necrosis factor antagonists in rheumatoid arthritis over 10 years: data from the Spanish registry BIOBADASER 2.0. Ann Rheum Dis 2012;71:382-5.
